# Nonsurgical Treatment of Maxillary Deficiency Using Tongue Guard Appliance: A Case Report

**DOI:** 10.5681/joddd.2011.031

**Published:** 2011-12-19

**Authors:** Farzaneh Ahrari, Neda Eslami

**Affiliations:** ^1^Orthodontist, Assistant Professor, Dental Research Center, Mashhad University of Medical Sciences, Mashhad, Iran

**Keywords:** Tongue guard, maxillary deficiency, orthodontic treatment, removable appliance

## Abstract

The present report describes orthodontic treatment of a patient with skeletal Class III malocclusion and maxillary hypopla-sia. To correct the retrusive maxilla, the treatment plan included an intra-oral removable device. It had the advantages of good patient cooperation, ease of construction and effective modification. Forward growth of the maxilla, minimal down-ward and backward rotation of the mandible and improved facial profile esthetics were achieved.

## Introduction

The prevalence of Class III malocclusion is 1–5% in United States and about 23% in Asian populations.^1^ In cases of Class III malocclusion due to maxillary deficiency, various treatment modalities can be used such as face mask appliance with dental^2^ or skeletal^3^anchorage, distraction osteogenesis,^4^ and orthognathic surgery.^5^However, the treatment of choice in growing children is growth modification to encourage maxillary advancement. It has been demonstrated that maxillary protraction is more effective in younger children than those in the older age range.^6^ The best treatment results can be obtained in the primary^7^and early mixed dentition^8^cases.

Although several studies have shown favorable skeletal changes in patients treated with a face mask appliance,^9 , 10^ this device is bulky, and poses difficulties in daily life of patients. In fact, achieving adequate patient compliance with face mask therapy is difficult in some children, if not impossible. If it were possible to treat a retrusive maxilla with a small intraoral appliance, this would be of great benefit for both patients and orthodontists. In this article, we report a case with a maxillary deficiency problem, treated with an intra-oral device referred to as “tongue guard” appliance.

### Appliance Design

Tongue guard appliance is a modified type of the tongue crib appliance, which is commonly used for habit breaking. This device was first applied by Jalali for treatment of maxillary deficiency cases referred to Mashhad Faculty of Dentistry.^11^ Tongue guard appliance has been in common use for more than 30 years in Mashhad Faculty of Dentistry with good treatment results and excellent patient cooperation. It can easily be constructed in the laboratory with low cost. Because of its intraoral use, patient compliance is rarely a problem with tongue guard appliance compared with extraoral protractors. This device transfers the tongue pressure to the cribs and finally to the upper jaw, resulting in effective forward movement of the maxilla and maxillary dentition.^12 , 13^ Unlike other treatment modalities that rely on backward mandibular rotation for correction of Class III malocclusions, mandibular rotation is not a mechanism of action for tongue guard appliance, making it useful in maxillary deficiency cases with long lower anterior face height. The best age range for tongue guard therapy is 6–8 years.

The tongue guard appliance (Figure 1, upper) consists of retention components (usually Adams clasps on permanent first molars and C clasps on deciduous canines), posterior bite planes and tongue guard (tongue cribs). These cribs are made of 0.8-mm stainless steel wire and have a height of 15–25 mm depending on patient’s facial height. They are imbedded in an acrylic plate about 15 mm behind maxillary anterior teeth, in a curved fashion. Usually a tongue guard consists of six loops, but in cases with lateral posterior open bite, an extended tongue guard can be employed to prevent placement of the tongue between the teeth and help tooth eruption. Posterior bite planes eliminate anterior teeth interferences, making crossbite correction fast and simple. These also impede eruption of posterior teeth and encourage eruption of anterior teeth to gain sufficient overbite, which is important for preventing relapse of anterior crossbite correction. The patient is asked to remove the appliance only during meals and while brushing.

In cases needing palatal expansion to correct the posterior crossbite, a median screw is incorporated in the plate and the tongue guard is made as two separate cribs (usually 3 loops are placed on each side of the split plate), which are individually embedded in acrylic plates (Figure 1, lower). Generally, the patient is instructed to open the screw once a week.


Figure 1. Tongue guard appliance (upper) and tongue guard appliance with a midpalatal screw and hooks for face mask therapy (lower).

**Figure:**
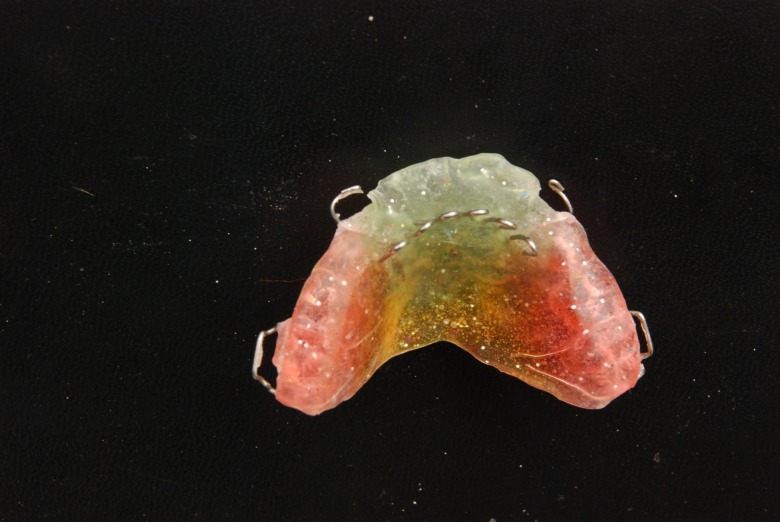

**Figure:**
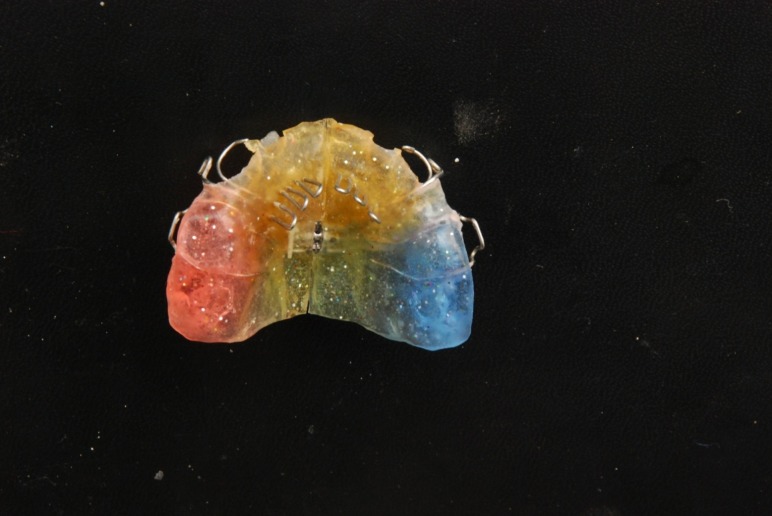

The important considerations in using the tongue guard appliance are correct construction and patient education. The tongue cribs should have sufficient height to prevent forward positioning of the tongue beneath the cribs that can exert forces to mandibular anterior teeth. If the tongue guard contacts the mouth floor, the loops can be bent inwards using Adams pliers to prevent soft tissue irritation. The patient should be instructed to place the tongue behind the cribs when swallowing, speaking, and more importantly at rest to transfer tongue pressure to the maxillary complex.

However, tongue guard therapy has one disadvantage. Since the tongue is placed behind the cribs at rest, equilibrium between tongue and lip pressures may change, resulting in lingual tipping and sometimes anterior crowding of mandibular incisors. This phenomenon can be prevented by inserting a lingual arch in patients with complete eruption of lower incisors to maintain lower arch periphery.^12^

## Case report

**Figure 2:**
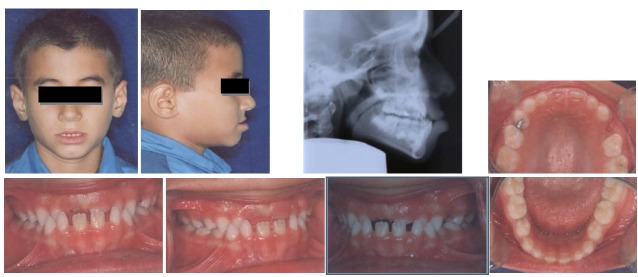
Pre-treatment extraoral and intraoral photographs along with the lateral cephalometric radiograph.

An 8-year-old boy referred to Mashhad Faculty of Dentistry with a chief complaint of reverse overjet. In clinical examination, the patient showed a concave profile, Class III molar and canine relationships and anterior crossbite (an overjet of −3 mm) (Figure 2). The cephalometric evaluation revealed that maxillary deficiency had a great contribution to his Class III malocclusion, although some mandibular prognathism was also evident in the lateral cephalogram (SNA=79°, SNB=80°, ANB=-1°). Vertical growth pattern of the patient was normal (FMA=25°, GoGn-SN=32°) (Table 1).

**Table 1:** Chephalometric measurements

Measurements	Pre-treatment 8 y 1 mo	Post-treatment 9 y 4 mo	Retention 12 y
SNA	79°	82°	82
SNB	80	80	80
ANB	-1	+2	+2
A–NPog (mm)	0	+1.5	+1.5
B–NPog (mm)	0	0	0
U_1_–SN	94°	99°	99°
U_1_–Pt.p	106°	108°	108°
U_1_–L_1_	146°	158°	159
U_1_–NPog (mm)	-1.5	+2	+2
L_1_–NPog (mm)	+4.5	-1	-0.5
Y-axis	63°	63°	63°
FMA	25°	25°	25°
GoGn–SN	32°	32°	32°
IMPA	88°	81°	80

The probability of a future maxillofacial surgery was explained to the patient and his parents, but they insisted on performing some form of treatment to at least reduce the severity of malocclusion. The patient refused to wear an extraoral device; therefore, a tongue guard appliance was prescribed for correction of Class III malocclusion.

**Figure 3:**
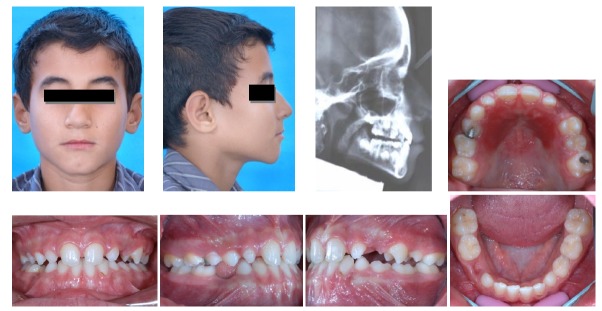
Post-treatment extraoral and intraoral photographs along with lateral cephalometric radiograph.

**Figure 4:**
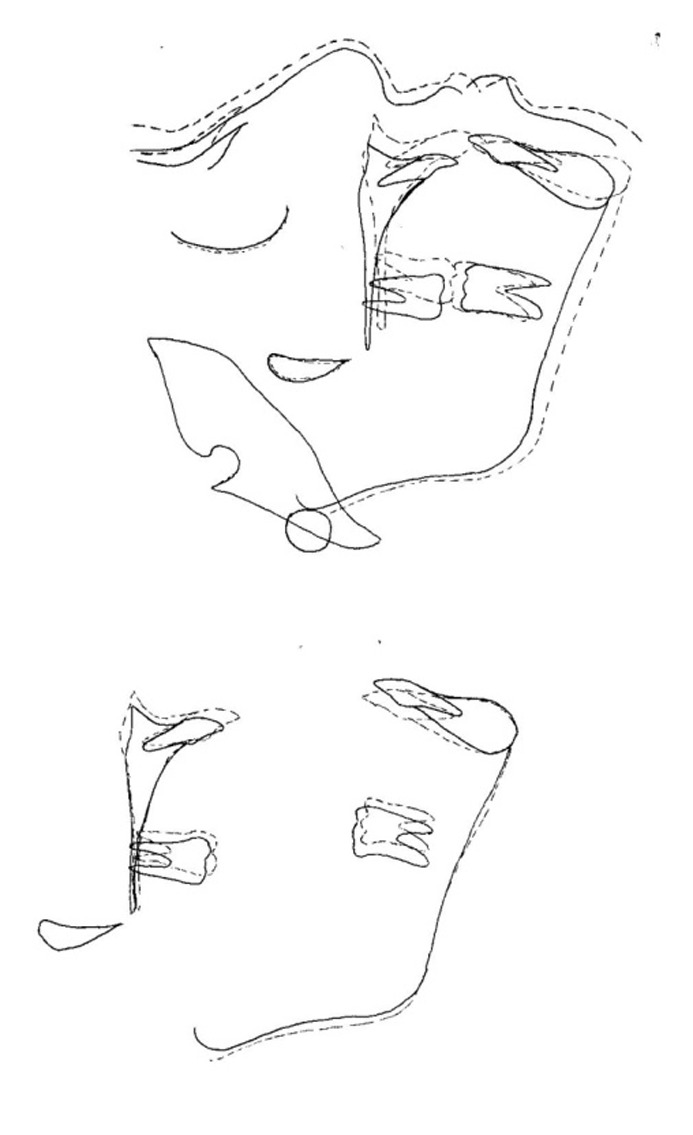
Superimpositions of cephalometric tracings before (solid line) and after (dotted line) treatment. (A) Superimposed on SN plane at S; (B) best fit superimposition of maxilla; (C) superimposed on mandibular plane at Me.

After 15 months of treatment with the tongue guard appliance, facial profile and lip closure dramatically improved and positive overjet was achieved (Figure 3). Cephalometric analysis revealed that SNA angle had increased from 79° to 82° and ANB angle had decreased from -1° to +2° during tongue guard therapy. Some forward tipping of maxillary incisors (U1 to SN: 94° to 99°) and some lingual tipping of mandibular incisors (IMPA: 88° to 81°) was also evident on superimposed tracings (Figure 4).

Although the facial height slightly increased during the treatment, post-treatment mandibular plane was parallel to the pretreatment one (FMA=25°, GoGn-SN=32°), implying that this change was essentially related to normal vertical growth of the face rather than to treatment result.

All the photographs are illustrated with written informed consent of the patient’s parents.

## Discussion

In the present study, forward and downward movement of maxilla was observed, evidenced by increased SNA and A-NPog. However, it should be reminded that proclination of upper incisors (increased U_1_-SN, U_1_-pt.p, U_1_-NPog) and retroclination of lower incisors (decreased IMPA) also contributed to the correction of reverse overjet. These results are consistent with the results of studies by Jalali and Poosti, using tongue guard appliance in patients with maxillary deficiency.^11 , 12 , 14^

Yavuz evaluated the effects of face mask therapy in two skeletally matured groups of female subjects with skeletal Class III malocclusion. The findings of the investigation are very similar to the results of this case.^15^

In the present case, minimal downward and backward rotation of the mandible was observed, which could be explained by normal vertical growth of the face. This was in contrast to the findings of Chong, who reported that downward and backward movement of the mandible and retroclination of mandibular incisors was among the major treatment effects of protraction headgear.^16^

Convincing an 8-year-old or even a younger child to wear a face mask appliance is a great challenge for orthodontists. The patient presented here wore the appliance 24 hours a day, except during meals and oral hygiene measures. However, we asked the patient not to remove the appliance during meals in the transitional stage of overjet correction when anterior teeth move from reverse overjet to positive overjet condition. This prevented incisor contacts and facilitated crossbite correction. Although some of the treatment results were due to forward movement of maxillary dentition and retroclintion of mandibular incisors, cephalometric superimpositions showed that forward and downward movement of the maxilla had a great contribution to treatment results.

The mechanism of action of this appliance can be attributed to the transmission of tongue pressures to the maxillary complex. According to Proffit et al, there are two types of tongue pressure: tongue pressure during swallowing and rest pressure of the tongue.^1^ The former is intermittent and may have little effect on Class III correction. Since the tongue is always placed behind the cribs, the rest pressure of the tongue contributes to Class III correction.

Posterior bite planes created the necessary clearance for forward movement of the maxillary dentition and helped increase the overbite. The latter is important in the treatment of reverse overjet cases, because it prevents the relapse tendency. Facial height slightly increased in the patient presented here. Since the mandibular plane remained relatively parallel to the pretreatment position, this movement was largely due to growth changes. Certainly, slight proclination of maxillary incisors and some retroclination of mandibular incisors contributed to overjet correction. However, these dental movements are also common consequences of treatment with extraoral maxillary protractors.^17^

Possibility of incorporating an expansion screw in this device to have simultaneous posterior and anterior crossbite correction is a great advantage. Several studies have shown that maxillary protraction is more effective when it is combined with maxillary expansion.^17^

It is important to note that tongue guard appliance can be used as a habit breaker in Class III patients with digit sucking habits. The cribs of this appliance do not allow the fingers to enter the mouth. In cases of Class I malocclusion, where maxillary forward movement is not desirable, interlocking of bite blocks can be used to stabilize maxillary and mandibular dentition.

In conclusion, tongue guard therapy can be considered the first treatment option in mild to moderate Class III cases exhibiting maxillary deficiency. In addition, when treatment with face mask appliance is planned, it is possible to incorporate hooks for face mask attachment in the tongue guard appliance (see Figure 1) and ask the patient to wear this plate all the time, even when an extraoral protractor is not used. Certainly, tongue guard appliance is a good alternative to face mask therapy in uncooperative Class III patients. In addition, this device can be used as a retainer to maintain the results of face mask therapy.

**Figure 5:**
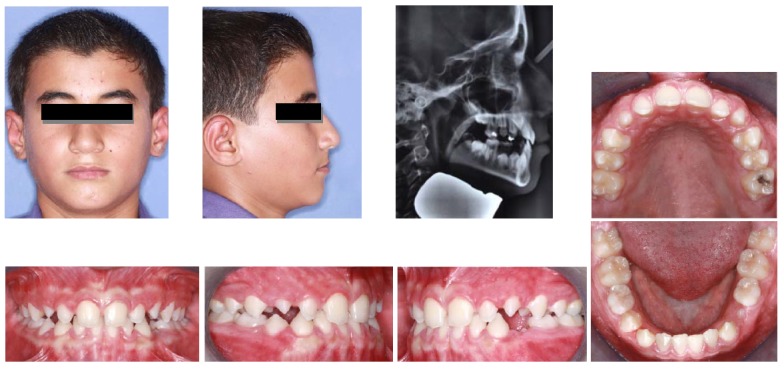
Post-treatment records four years after tongue guard therapy.

Night-time wear of a tongue guard appliance, as a retention protocol, is strongly suggested after active treatment. Figure 5 illustrates the patient’s records four years after starting tongue guard therapy. As shown in the photographs, long-term stability of treatment results has been achieved.

Further research on the effects of this appliance is required to assess its long-term effects.

## Conclusion

Tongue guard appliance is effective for correction of mild to moderate skeletal Class III malocclusions, resulting in forward movement of maxillary complex.
